# A new method of intermittent lower dose of tolvaptan combined with fluid restriction to treat the syndrome of inappropriate antidiuresis: A case report

**DOI:** 10.1097/MD.0000000000017586

**Published:** 2019-10-25

**Authors:** Xianxian Yuan, Hui Pan, Huijuan Zhu, Jiapei Li, Hui Miao, Xiaoan Ke, Shi Chen

**Affiliations:** Department of Endocrinology, Peking Union Medical College Hospital, Chinese Academy of Medical Science and Peking Union Medical College, Key Laboratory of Endocrinology of National Health Commission of the People's Republic of China, Beijing, China.

**Keywords:** fluid restriction, the syndrome of inappropriate antidiuresis, tolvaptan

## Abstract

**Rationale:**

Tolvaptan, an oral vasopressin V_2_ receptor antagonist, is a new approach for the treatment of adult patients with the syndrome of inappropriate antidiuresis (SIADH). However, dose-dependent side effect including rapid increase in serum sodium levels and liver injury, and the expensive price limit the long-term use of tolvaptan. We report a case of SIADH patient treated with intermittent lower dose of tolvaptan combined with fluid restriction.

**Patient concerns:**

A 60-year-old woman presented of nausea and vomiting, dizzy and amaurosis, and transient disturbance, after a week of persistent diarrhea.

**Diagnosis:**

Diagnosis of SIADH was based on severe persistent hyponatremia, decreased plasma osmolality, raised urinary sodium excretion, and the absence of other causes.

**Interventions:**

She was given the treatment of tolvaptan 15 mg once daily, and experienced tolvaptan-related side effects including thirst and dry mouth, polyuria, and dizziness. Then, single dose of tolvaptan was reduced from 15 to 7.5 mg, and the interval between medication was gradually prolonged from 24 to 72 hours. Meanwhile, serum sodium was negatively correlated with the amount of daily water intake in interval days, so daily water intake of the patient was restricted to 1500 mL in interval days.

**Outcomes:**

Serum sodium was maintained within the normal range, 137 to 141 mmol/L without liver damage.

**Lessons:**

For patients with chronic SIADH, the tolvaptan dose should be individualized, and the regimen of intermittent lower dose of tolvaptan combined with fluid restriction maybe an effective choice.

## 1 Introduction

Chronic hyponatraemia caused by the syndrome of inappropriate antidiuresis (SIADH) not only induces uncharacteristic clinical symptoms but also increases morbidity and mortality, which requires long-term treatment.^[1]^ Tolvaptan, an oral vasopressin V_2_ receptor antagonist, was approved by the United States and Europe for the treatment of adult patients with hyponatremia secondary to SIADH in 2009.^[2]^ However, the United Kingdom Medicines and Healthcare Products Regulatory Agency in 2012 warned that tolvaptan may cause rapid increases in serum sodium levels and serious neurological events.^[3]^ It was also reported to be associated with dose-dependent liver injury,^[4]^ and the USA Food and Drug Administration (FDA) recommended that tolvaptan should not be used for >30 days and should not be used in patients with underlying liver disease.^[5]^ Moreover, because tolvaptan is not reimbursed by the Chinese Government through the Pharmaceutical Benefits Scheme, the cost to patients who need long-term tolvaptan treatment is another important consideration. Therefore, it is necessary to find a safe, effective, and economical method of using tolvaptan for chronic hyponatreamia patients. We report a case of SIADH patient treated with intermittent lower dose of tolvaptan combined with fluid restriction which may reduce tolvaptan-related side effects and finance burden for SIADH patients who need long-term treatment.

## 2 Case presentation

**Figure 1:**
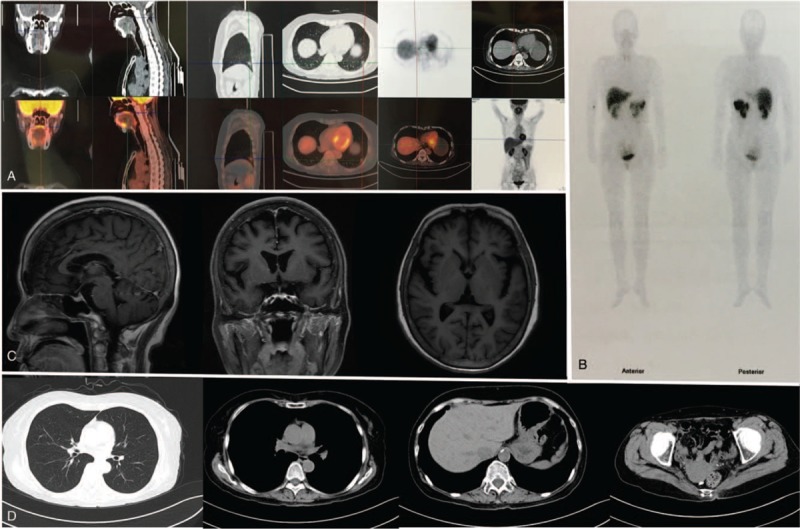
Imaging materials of the patient. (A) ^18^F-PET/CT. (B) ^99m^Tc-HTOC-SPETCT. (C) Magnetic resonance imaging of craniocerebral. (D) Computed tomography of chest, abdomen, and pelvis.

A 60-year-old woman was admitted to the Department of Endocrinology because of persistent hyponatremia. Five months before admission, the patient complained of nausea and vomiting, dizzy and amaurosis, and transient disturbance with normotension (118/80 mm Hg), after a week of persistent diarrhea. Biochemical test showed hyponatremia and hypochloremia without hyperglycaemia and hyperlipidemia (as shown in Table 1), and the minimum of serum sodium and serum chlorinum were 119.0 and 82.3 mmol/L, respectively. Meanwhile, laboratory tests showed effective serum osmolality 256.3 mOsm/kg, urine specific gravity 1.025, urine sodium 134.2 mmol/L, uric acid 195.0 mmol/L, and urea nitrogen 4.8 mmol/L. There was no evidence for adrenal, thyroid, pituitary and renal insufficiency, and no recent use of diuretic agents (as shown in Table 1). Her serum sodium was still 121.9 mmol/L after 0.9% saline infusion, but rose with fluid restriction to 128 mmol/L. Thus, the diagnosis of SIADH was confirmed according to the diagnostic criteria,^[6]^ but the cause was not clear, as there was no evidence for drugs, malignant diseases, pulmonary disorders, and disorders of the nervous system (as shown in Fig. 1).

**Table 1:**
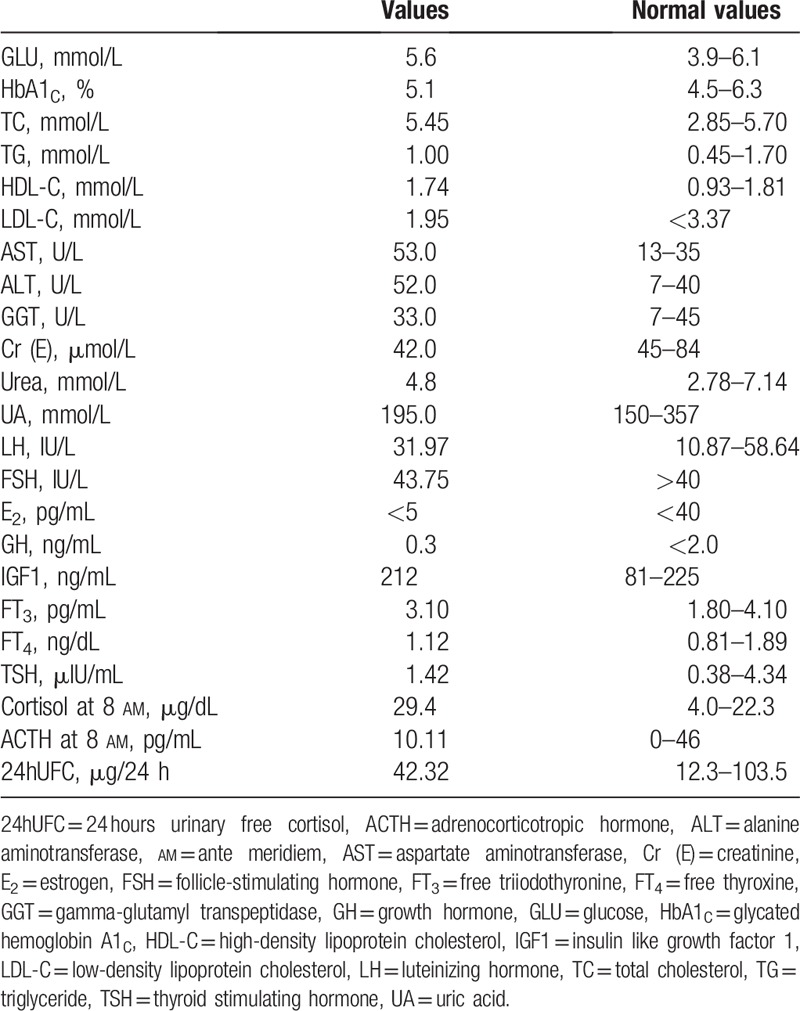
Biochemical and hormonal findings of the patient.

**Figure 2:**
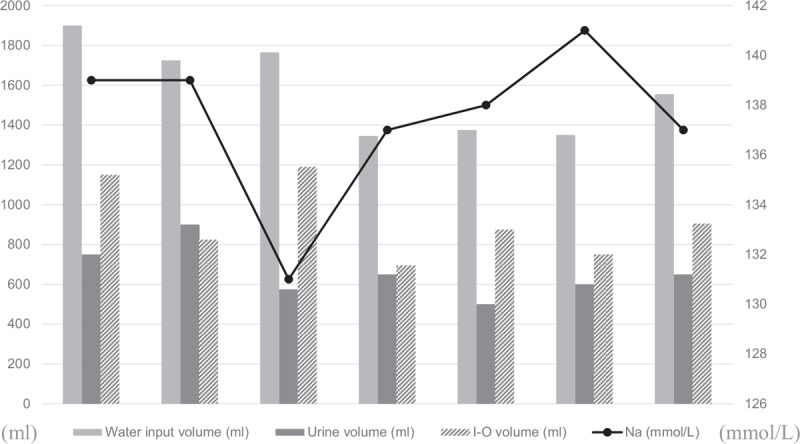
Serum sodium, water input volume, and urine volume of the patient. The water input volume and urine volume were recorded between tolvaptan doses. Serum sodium was detected before the next tolvaptan dose.

Because the patient had a low daily water intake and could not tolerate fluid restriction, hypertonic saline solution was given to her and serum sodium fluctuated between 119.6 and 130.6 mmol/L. Therefore, she was given the treatment of tolvaptan 15 mg per 24 hours, her serum sodium increased from 130.0 to 145.8 mmol/L, urine sodium decreased from 102.5 to 37.3 mmol/L, and urine specific gravity decreased from 1.020 to 1.005. Not surprisingly, she experienced the most common tolvaptan-related side effects, including thirst, urinary frequency and polyuria, and dizziness. Meanwhile, serum aminotransterases were slightly increased, alanine aminotransterase (ALT) 56 U/L and aspartate aminotransferase (AST) 53 U/L. Taking tolvaptan-related side effects and finance burden into account, we carefully titrated the dosage and interval of tolvaptan under careful measurement of serum sodium concentration after admission. The dosage of tolvaptan was gradually reduced from 15 mg per 24 hours to 7.5 mg per 72 hours. Meanwhile, we found that serum sodium was negatively correlated with the amount of daily water intake in interval days (as shown in Fig. 2). Daily water intake of the patient was restricted to 1500 mL in interval days. There was no limit to the amount of water intake on the day of taking tolvaptan to prevent rapid increase in serum sodium levels and relieve tolvaptan-related discomforts including thirst and dry mouth. After titrating, the regimen of tolvaptan 7.5 mg per 72 hours and water intake volume <1500 mL/d in interval days was appropriated for the patient to maintain serum sodium in normal range (137–141 mmol/L) without liver damage (ALT 33 U/L and AST 32 U/L).

## 3 Discussion

SIADH is a common cause of chronic hyponatremia, which may adversely affect health status, causing, for instance, gait disturbances, attention deficit, falls, and fractures, especially in the elderly.^[1]^ Fluid restriction is regarded as first-line treatment for moderate or profound hyponatremia due to SIADH.^[6]^ But it is not practical in all patients, especially in the elderly, as lower water intake is not tolerated. Urea is an alternative effective treatment for SIADH; however, the unpleasant taste has limited its use and it is unavailable in China. Tolvaptan is a nonpeptide, selective arginine vasopressin V_2_ receptor antagonist that increases free water clearance, thereby correcting low serum sodium levels,^[2]^ which has been confirmed to be effective in hyponatremia caused by SIADH.^[7,8]^

Tolvaptan treatment is to be initiated at 15 mg once daily with the dose increased to a maximum of 60 mg once daily. Adverse effects of tolvaptan include thirst, urinary frequency, dehydration, and too-rapid correction of sodium which is dose related.^[2,3]^ Rapid correction of serum sodium in patients with chronic hyponatremia can lead to neurologic sequelae, including osmotic demyelination. Moreover, tolvaptan was reported to be potentially at increased risk of liver injury that had been warned by FDA,^[4,5]^ which was dose related and reversible after withdrawal of tolvaptan. A clinical trial of tolvaptan in patients with autosomal dominant polycystic kidney disease has identified an increased risk of serious liver injury in patients assigned 120 mg tolvaptan daily compared with placebo,^[4]^ which were generally higher than that in SIADH patients. Another important concerns of using tolvaptan is its cost, which is not reimbursed by the Chinese Government through the Pharmaceutical Benefits Scheme. In China, a tablet (15 or 30 mg alike) is presently sold for RMB ¥ 180, and a month of tolvaptan treatment could cost as much as RMB ¥ 5400, roughly equivalent to US $ 793, which was a heavy financial burden for patients who need long-term tolvaptan treatment. There is no published prospective evidence demonstrating how such costs can be justified in terms of benefit to the patient and to the healthcare system.

Thus, it is necessary to find a safe, effective, and economical method of using tolvaptan for chronic hyponatremia patients, and individual-based tolvaptan therapy should be emphasized. A lower starting dose 7.5 mg once daily was also recommended on a patient-by-patient case basis and clinical trial.^[9]^ Once-weekly 15 mg tolvaptan for chronic symptomatic hyponatremia due to SIADH in an 83-year-old man was reported, whose serum fluctuated between 122 and 133 mmol/L.^[10]^ A lower single dose of tolvaptan in our patient could prevent too-rapid correction of sodium and significantly relieve tolvaptan-related discomforts such as thirst, dry mouth, and dizziness. Combined with fluid restriction, interval between dosing of tolvaptan could be prolonged, and the dosage and cost of tolvaptan decreased significantly. In turn, intermittent dosing of tolvaptan could prevent dehydration and discomforts secondary to fluid restriction. In the treatment of intermittent lower dose of tolvaptan, serum aminotransterases of our patient gradually decreased to normal range. Whether hepatotoxicity of tolvaptan is similar when used intermittently in lower doses for longer periods is unclear, so regular examination of liver function is mandatory.

In conclusion, for patients with chronic SIADH, in our experience, the tolvaptan dose should be individualized and could be progressively reduced once adequate sodium is achieved. The regimen of intermittent lower dose of tolvaptan combined with fluid restriction in interval days was effective and acceptable. Decisions with regard to longer term treatment for chronic SIADH need to consider a balance between effects and side effects, and that between the ongoing cost of therapy and the patient's benefit, which need to be addressed on an individual basis. Prospective case control study is needed to compare the regimen in our report and conventional regimen in terms of effectiveness, safety, acceptance, and cost.

## Acknowledgments

The study was approved by the Ethics Committee of the Peking Union Medical College Hospital (PUMCH; No. JS1233). Written informed consent was obtained from the patient, including the permission for details and images relating to the patient to be published. The patient was informed that the details and images would be freely available on the Internet and may be seen by the general public. Copies of the consent forms are available for review by the editor of this journal.

## Author contributions

**Conceptualization:** Xianxian Yuan, Shi Chen.

**Data curation:** Jiapei Li, Hui Miao, Xiaoan Ke.

**Formal analysis:** Xianxian Yuan, Shi Chen.

**Investigation:** Jiapei Li.

**Methodology:** Shi Chen.

**Project administration:** Hui Pan, Huijuan Zhu, Shi Chen.

**Writing – original draft:** Xianxian Yuan, Shi Chen.

**Writing – review and editing:** Xianxian Yuan, Shi Chen.

Xianxian Yuan orcid: 0000-0001-8762-8471.
